# Problem-based learning and undergraduate research: another student’s perspective

**DOI:** 10.1007/s40037-013-0085-9

**Published:** 2013-09-27

**Authors:** Joule J. Li

**Affiliations:** School of Medicine, Faculty of Health Sciences, University of Adelaide, c/o Medical School South Building (Room S511a), Frome Road, Adelaide, SA 5005 Australia

Dear Sir,

I thank Dr. AlAmodi for his thought-provoking *Eye Opener* on the similarity between problem-based learning (PBL) and undergraduate research [1]. Overall, I agree with Dr. AlAmodi’s ‘small laboratory’ concept, and based on my own experience of a case-based learning (CBL) curriculum, I believe the concept extends beyond PBL to similar small-group inquiry-based teaching methods such as CBL. Hypothesis-driven reasoning mirrors the scientific method whether it is guided (CBL) or open (PBL) inquiry. Indeed, I agree with Dr. AlAmodi that the generation of differential diagnoses in CBL/PBL mirrors the generation of specific research questions and the testing of these differential diagnoses against subsequent history, examination, and investigation results is akin to the statistical testing of research questions against collected data. However, I believe there are further similarities between CBL/PBL and research that are also noteworthy.

Unlike in traditional lecture-based curricula where specific study material is usually set by the course coordinator and/or lecturers, study for CBL/PBL sessions is self-directed [2]. As a result of this self-directedness, in my experience, medical students utilize multiple different online and paper-based information sources to prepare for CBL/PBL sessions. This, in turn, requires students to develop skills in synthesizing and critically analyzing large amounts of information, a concept known as ‘information literacy’ [3]. The information literacy skills developed as a result of CBL/PBL are highly transferable to the research context, which is also self-directed and involves critically reviewing large amounts of literature.

Furthermore, the communication and teamwork skills that result from CBL/PBL [2] are relevant to conducting research. Communication skills developed in CBL/PBL are similar to those required in research. For example, the ability to justify one’s contributions to CBL/PBL discussions by referencing citable sources of information is similar to the ability to defend one’s research methods, results, and conclusions. The teamwork skills developed in CBL/PBL, such as the ability to amicably resolve differences of opinion, are also transferable to the team-based and collaboration-based research contexts.

The pedagogies of CBL/PBL and research require students to acquire similar skills and utilize similar methods of cognitive reasoning. It would be interesting to investigate whether students of CBL/PBL curricula become more active researchers than students of traditional lecture-based curricula.
